# Identifying the amino acid domains of ORF59 responsible for interactions with ORF57 and PAN RNA during KSHV lytic replication

**DOI:** 10.1128/spectrum.01163-24

**Published:** 2024-10-21

**Authors:** Shannon Harger Payen, Isaura Vanessa Gutierrez, Kayla Andrada, Subhash C. Verma, Cyprian C. Rossetto

**Affiliations:** 1 Department of Microbiology and Immunology, University of Nevada, Reno School of Medicine, Reno, Nevada, USA; Oklahoma State University College of Veterinary Medicine, Stillwater, Oklahoma, USA

**Keywords:** KSHV, viral DNA replication, virus interactions, protein-protein interactions, RNA-protein interactions

## Abstract

Kaposi’s sarcoma-associated herpesvirus (KSHV) DNA polymerase processivity factor, ORF59, is a lytic protein essential for viral DNA synthesis as part of the core replication complex. The multifunctional nature of ORF59 has prompted the investigation into its various functional domains. Prior studies of ORF59 have identified dimerization, DNA interaction, and polymerase interaction domains. The regions of ORF59 responsible for the interaction with the viral mRNA transport accumulation protein (MTA/ORF57) and the viral long non-coding polyadenylated nuclear (PAN) RNA have not been explored. Using a series of previously characterized ORF59 deletion KSHV BACmid mutants, we identified the domains of ORF59 that interact with ORF57 and PAN RNA. Interestingly, amino acids 51–100 were essential for interacting with both ORF57 and PAN RNA. Using this information, we generated a plasmid that expresses a DsRed-tagged polypeptide spanning amino acids 30–100 of ORF59. When the 30–100 aa DsRed-tagged polypeptide expression plasmid was transfected into KSHV wild-type iSLK cells prior to lytic reactivation, a dominant-negative inhibition of virus replication was observed, resulting in a decrease of infectious virus production. Our data suggest that interactions between ORF59 with ORF57 and PAN RNA are important to successful lytic replication.

**IMPORTANCE:**

To better understand the Kaposi’s sarcoma-associated herpesvirus (KSHV) DNA polymerase processivity factor ORF59, we investigated the interaction of ORF59 with ORF57 and polyadenylated nuclear (PAN) RNA. We used a previously characterized KSHV BACmid containing internal deletions of ORF59 to identify the domains of ORF59 that interact with ORF57 and PAN RNA. Our study revealed multiple domains of ORF59 that are essential for its association with PAN RNA. These domains span amino acids 51–100, 251–300, and 351–396. Additional experiments confirmed amino acids 51–100 are critical for the interaction between ORF59 and ORF57. Using this information, we generated an expression plasmid encompassing the ORF57 and PAN RNA interaction domains of ORF59. The ORF59 polypeptide expression plasmid of amino acids 30–100 functioned as a dominant negative inhibitor during viral reactivation and caused a decrease in virus production. These findings provide valuable insights into the key domains of ORF59, essential for its functionality, and ultimately the production of infectious viruses.

## INTRODUCTION

Viruses in the *Herpesviridae* family contain linear double-stranded DNA genomes encased in a nucleocapsid and an envelope (1). Members of the *Herpesviridae* family share a common mammalian herpesvirus ancestor and are further divided into three subfamilies: *Alpha*-, *Beta*-, and *Gammaherpesvirinae* (*α-*, *β-*, and γ-) (2, 3). Kaposi’s sarcoma-associated herpesvirus (KSHV), or human herpesvirus 8, is a γ-herpesvirus that replicates in lymphoblastoid cells, mainly B lymphocytes, but is known to infect other cell types such as fibroblasts, endothelial, and epithelial cells (3). One clinical manifestation of KSHV is Kaposi’s sarcoma (KS), a tumor disease of endothelial cell origin. It was first described in 1872 by Moritz Kaposi, but KSHV was not isolated and attributed to KS until 1994 (4). KSHV is an oncogenic virus and, in addition to KS, is also the causative agent of multicentric Castleman’s disease and primary effusion lymphoma (5–7). Additionally, patients with KS can develop cytokine syndrome or Kaposi’s Sarcoma Inflammatory Cytokine Syndrome when there is an elevated viral load (8).

KSHV, like all herpesviruses, has a bi-phasic replication cycle consisting of latency and reactivation (9). During latency, few viral genes are expressed, and the viral genome is maintained as a circular episome tethered to host chromosomes by open reading frame 73, the latency-associated nuclear antigen (10, 11). The switch from latency to reactivation kicks off a cascade of viral gene expression beginning with immediate early (IE), to early (E), and finally late (L) genes (12). The DNA polymerase processivity factor, ORF59 or PF-8, is an early viral protein (13). ORF59 assists the viral DNA polymerase ORF9/Pol-8 by binding to it in the cytoplasm and shuttling it to the nucleus where it can interact with other replication proteins at the origin of lytic DNA replication (*ori*Lyt) to begin viral DNA synthesis (14). KSHV encodes six core replication proteins required for DNA synthesis. The replication complex consists of ORF6 (single-stranded DNA-binding protein), ORF9 (DNA polymerase), ORF40/41 (primase-associated factor), ORF44 (helicase), ORF56 (primase), and ORF59 (polymerase processivity factor) (12, 15–17). In addition to the core replication complex, viral initiation and accessory factors such as the replication and transcription activator (K-RTA encoded by ORF50) and K-bZIP (K8) are recruited to *ori*Lyt (14, 15). Homologs of ORF59 in other human herpesviruses include herpes simplex-1 (HSV-1) UL42, Epstein-Barr virus (EBV) BMRF1, human cytomegalovirus (HCMV) UL44, and human herpesvirus-6 p41 (18).

Although ORF59 has primarily been investigated as a processivity factor, recent studies have begun to reveal diverse functions of ORF59. The variety of functions is reflected by the abundant binding partners of ORF59, including viral and cellular factors as well as the highly abundant KSHV long non-coding (lnc) polyadenylated RNA, polyadenylated nuclear (PAN). Recently, ORF59 has been shown to have interactions with cellular proteins that influence replication and transcription including histones and histone demethylases (19, 20). Interestingly, a prior proteomics analysis of ORF59 binding partners during lytic reactivation identified viral proteins including the early viral mRNA transcript accumulation (MTA, encoded by ORF57) protein (21). Homologs to KSHV ORF57 include HSV-1 ICP27, EBV BMLF1 (SM), varicella zoster virus ORF4, HCMV UL69, and herpesvirus saimiri ORF57. The viral MTA protein is a multifunctional regulator involved in facilitating lytic gene expression and primarily functions to enhance the stability of intronless viral RNA within host cells to promote efficient translation of viral transcripts (22). ORF57 has also been shown to interact with cellular and viral non-coding RNAs including spliceosome U1 and KSHV PAN RNA (23, 24). ORF57 interacts with PAN RNA at the 5′-region named the MTA-responsive element and stabilizes the PAN transcript. Stabilization of PAN RNA by ORF57 allows for the high accumulation of PAN (25, 26). A series of studies have indicated that ORF57 promotes the expression of intronless RNA but does not exhibit a directed RNA export function (27). ORF57 is an essential protein, and in the context of virus replication, a deletion mutant of ORF57 had a defect in lytic viral gene expression and could not produce an infectious virus (28).

Interestingly, both ORF59 and ORF57 interact with PAN RNA (29). PAN RNA is the most abundant transcript during lytic replication. It is initially expressed during the early phase, and its levels are maintained throughout the late stages (30). The high abundance is attributed to the highly active K-RTA response element within the PAN promoter in addition to the stability of the transcript provided by ORF57 (31). PAN is a multifunctional lncRNA and has been suggested to promote viral gene expression by interactions with histone modification complexes (32, 33). PAN RNA is required for the successful completion of viral replication, as both deletion and knockdown studies have shown PAN RNA as an essential viral RNA (34). Because of the previous reports of the interactions between ORF59 and ORF57, ORF59 with PAN RNA, and ORF57 with PAN RNA, we hypothesized that the interaction between these three viral factors may be required for replication.

To better understand the functional significance of the interaction between ORF59, ORF57, and PAN RNA, we focused on identifying the domains of ORF59 that interact with ORF57 and PAN RNA. We conducted experiments using iSLK cells harboring either wild-type (WT) KSHV or mutant BACmids with sequential 50-amino acid (aa) deletions in the ORF59 protein. We found that the ORF59 Δ51–100 mutant was unable to interact with ORF57 and PAN RNA. Using this information and taking into consideration the three-dimensional structure of ORF59, we created a dominant negative peptide spanning amino acids 30–100 of ORF59. This small polypeptide was able to bind to ORF57 and PAN RNA, blocking the interaction with full-length ORF59. The transfection of the ORF59 30–100 aa expression plasmid into iSLK cells containing WT KSHV prior to reactivation significantly decreased viral DNA synthesis and infectious virus production. These findings suggest that the interaction of ORF59 with ORF57 and PAN RNA is important for successful lytic replication.

## RESULTS

### Summary of previously identified functional domains of ORF59

**Fig 1:**
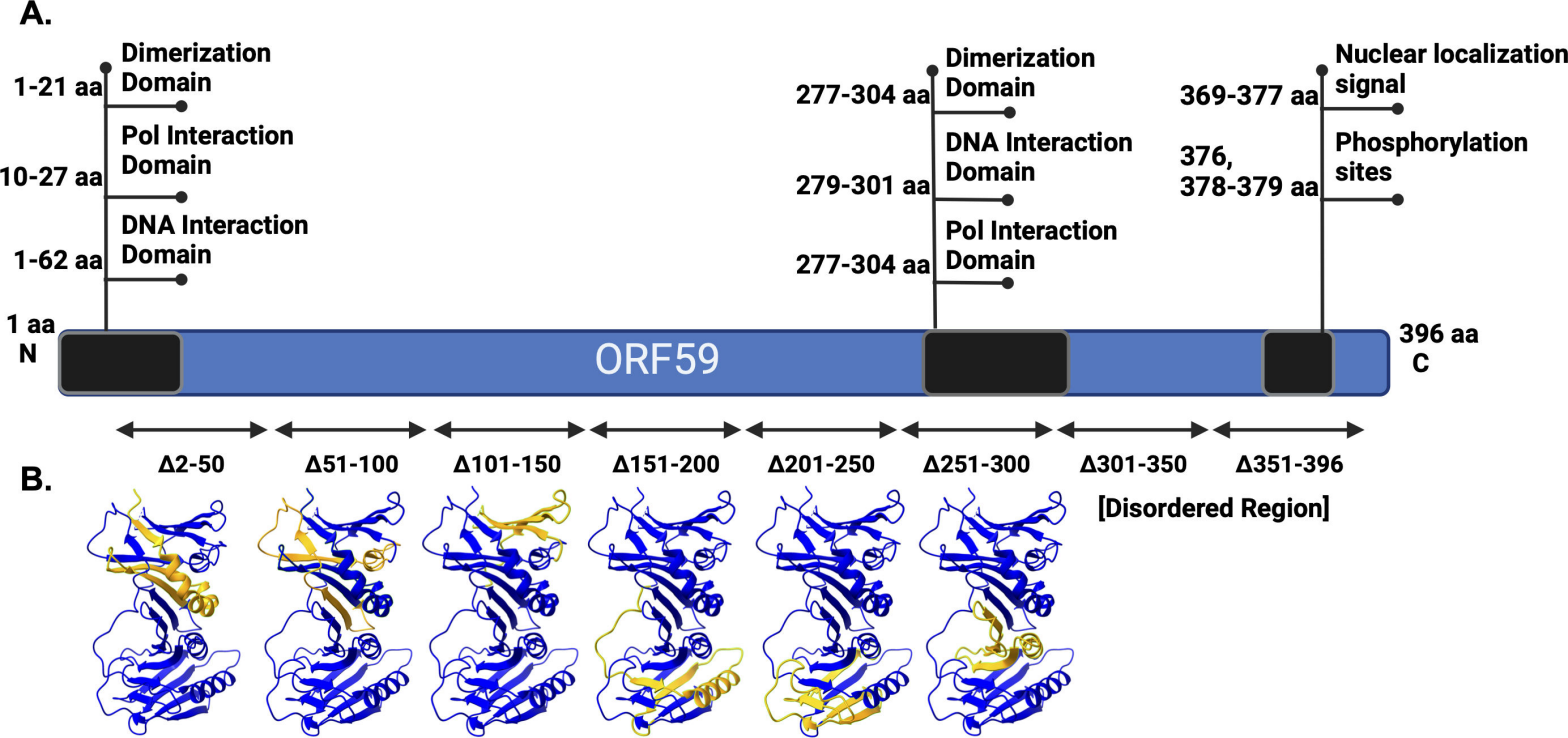
Schematic diagram of ORF59, the functional regions, and the deletion mutants used in this study. (**A**) Previously identified protein-protein and/or protein-DNA interaction domains of ORF59 (black). The identified domains include dimerization at 1–21 aa and 277–304 aa, DNA polymerase interaction (ORF9, Pol-8) at 10–27 aa, 277–304 aa, DNA interaction at 1–62 aa, 279–301 aa, NLS between 369 and 377 aa, and phosphorylation sites at aa 376 and 378–379. (**B**) A series of 50-aa deletion mutants in ORF59 used in this study. Below the linear representation are the crystal structures (PDB ID: 3HSL) of the ORF59 monomer (blue) with each deletion region highlighted in yellow. There is no crystal structure for the disordered region at the C-terminus.

Many prior investigations have contributed to our understanding of the functional domains of ORF59 (18, 21, 35). The immediate N-terminus contains regions that are essential for ORF59 self-dimerization, interaction and dimerization with ORF9/Pol-8, and interaction with DNA(36–38). Additionally, toward the C-terminus amino acids 277–304, there are similar interaction regions for self-dimerization, ORF9 dimerization, and interaction with DNA (18). The C-terminus also contains the proposed nuclear localization sequence (NLS) between aa 369 and 377 and multiple phosphorylation sites at serine 376, 387, and 379 (37). Previously identified functional domains are summarized in Fig. 1A. The region of ORF59 between amino acids 266 and 396 has been shown to interact with the major viral transactivator, K-RTA (14, 37). Previous research from our lab showed that multiple regions of ORF59 interact with canonical histones H2A, H2B, H3, and H4, along with the linker H1 (19).

To gain further insights into the domains of ORF59 that interact with ORF57 and PAN RNA, we used a series of ORF59 deletion BACmids to identify regions of ORF59 that are required for the interaction with ORF57 and PAN RNA. The ORF59 mutant BACmids containing internal deletions of ORF59 were previously described and characterized (19). Briefly, we generated eight sequential 50 amino acid deletions in ORF59, starting from amino acids 2 through 396, with an in-frame hemagglutinin (HA) tag (schematically shown in Fig. 1A). These mutant BACmids were used to establish latent KSHV in iSLK cell lines which could be reactivated with a combination of Doxycycline (Dox), 12-O-Tetradecanoylphorbol-13-acetate (TPA), and sodium butyrate (NaB) to test for defects in reactivation and lytic replication related to the function of ORF59 (19). While all the ORF59 deletion mutants could undergo reactivation and proceed through IE and into E phases, as demonstrated by the expression of K-RTA and ORF59, only those with the far C-terminal deletions were still able to synthesize viral DNA and produce an infectious virus, albeit to a substantially reduced amount (19). Because there were no apparent defects in early replication kinetics associated with the previously reported ORF59 deletion mutants, we used the mutants to help identify the domains of ORF59 that interact with ORF57 and PAN RNA during the early phase of reactivation.

To further understand the three-dimensional structure of ORF59 and visualize the physical domains encompassing each ORF59 deletion region, we generated three-dimensional protein structures using the ORF59 crystal structure (PDB ID: 3HSL) and UCSF ChimeraX (39). The region for each of the 50 amino acid deletions is highlighted in yellow from the remaining full-length ORF59 indicated in blue (Fig. 1B). The disordered region within the C-terminus is excluded because of the lack of a defined crystal structure.

### Multiple regions of ORF59 associated with PAN RNA

**Fig 2:**
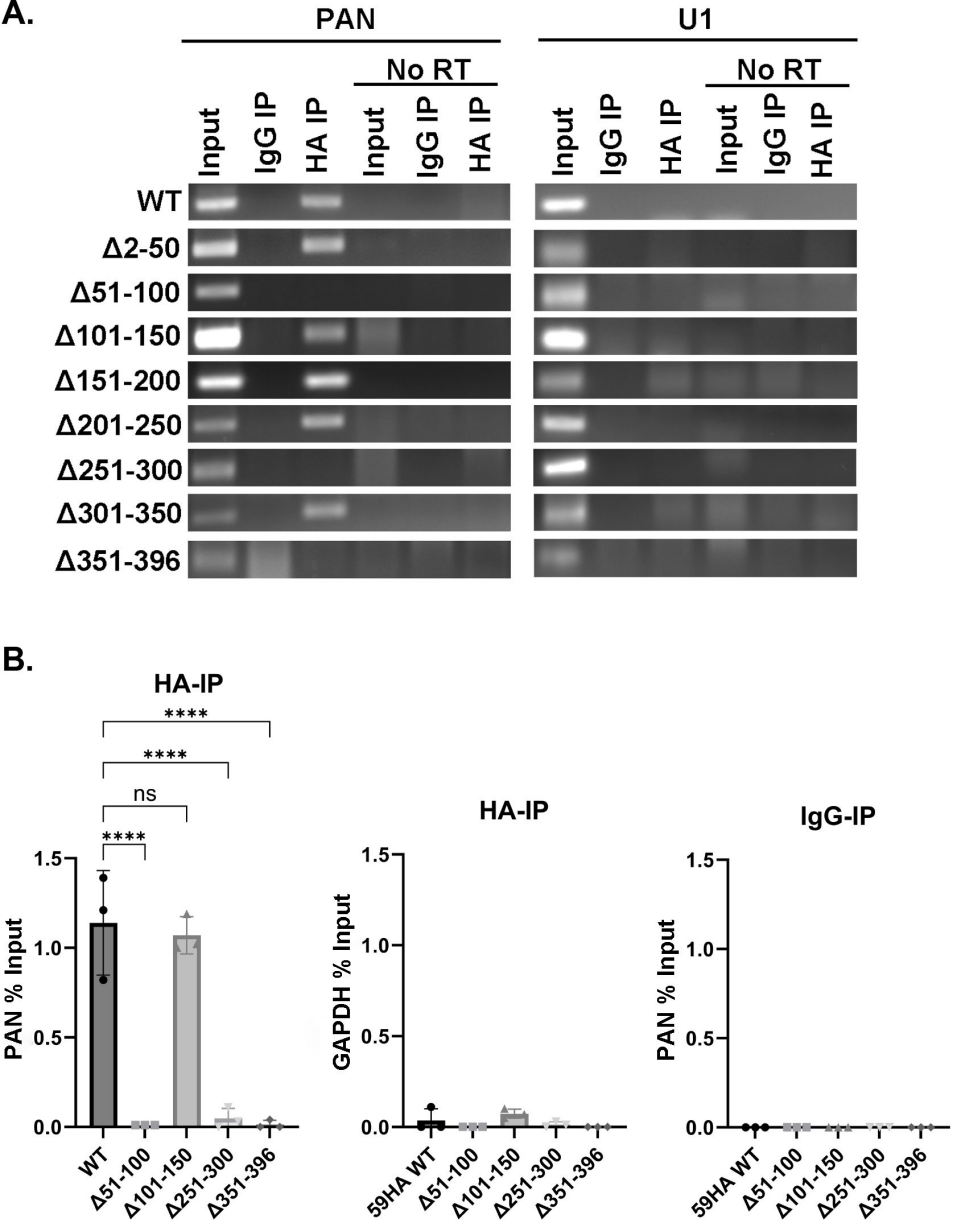
RNA-immunoprecipitations of 59HA-WT and deletion mutants demonstrate regions 51–100, 251–300, and 351–396 are necessary for ORF59 association with PAN RNA. (**A**) iSLK cells harboring either 59HA-WT or ORF59 deletion mutants were induced for 24 hours with Dox, TPA, and NaB. Protein-RNA complexes were isolated using anti-HA antibodies for ORF59HA or anti-IgG isotype control antibodies. The RNA was treated with DNase followed by cDNA synthesis with RT-PCR, and samples that did not receive reverse transcriptase (“no RT”) were used as control. Primers for PAN RNA or cellular U1 (control) were used for RT-PCR, and the resulting DNA products were resolved by agarose gel electrophoresis. (**B**) The experiments described above were repeated with the ORF59 deletion mutants that no longer interacted with PAN RNA (Δ51–100, Δ251–300, and Δ351–396) and assessed by RT-qPCR assay, along with WT and one deletion mutant that still retained the ability to interact with PAN RNA (Δ101–150). Percent input from each of the RIPs was calculated, and bar graphs represent means ± SDs. An ordinary one-way ANOVA (analysis of variance) nonparametric Brown-Forsythe test was performed (*P* value of <0.05). ****P = 0.0001.

Our first goal was to determine the 50 amino acid region of ORF59 that interacts with PAN RNA during lytic replication. An initial examination was done using RNA-immunoprecipitation (RIP) with reverse transcription-polymerase chain reaction (RT-PCR followed by agarose gel electrophoresis analysis of the resulting products from the input, HA-RIP, and IgG-RIP RNA (Fig. 2A). The iSLK.BAC16-ORF59HA (59HA-WT) and the eight sequential 50 aa deletions BACmids cells were treated for 24 hours with Dox, TPA, and NaB to trigger the reactivation of the KSHV lytic cycle. After 24 hours, cells were lysed. The lysate was incubated with anti-HA-antibody and magnetic protein G beads to isolate ORF59HA full-length or ORF59HA deletion proteins and the associated RNAs. RT-PCR was performed with primers to amplify either PAN RNA or cellular U1 RNA. Cellular U1 spliceosomal RNA is a small nuclear RNA and is one of the most abundant cellular non-coding RNAs (40). An IgG isotype control and no reverse transcriptase (No-RT) control were used for non-specific interactions and DNA contamination, respectively. RT-PCR gels revealed the ORF59 Δ51–100, Δ251–300, and Δ351–396 mutants were unable to interact with PAN RNA (Fig. 2A). Because RT-PCR followed by agarose gel electrophoresis provides only qualitative data, it was insufficient to determine potential minor differences among ORF59 Δ51–100, Δ251–300, and Δ351–396 deletion mutants in PAN RNA interaction capabilities. However, the experiment was repeated and evaluated through RT-qPCR.

To obtain a more quantitative measurement of the amount of ORF59 binding with PAN RNA, we performed the same RIP assay as above but used RT-qPCR to determine if there were any relative binding differences between the deletion mutants which no longer showed interaction with PAN RNA by RT-PCR. In addition, we selected one deletion mutant (ORF59 Δ101–150) that still retained the ability to interact with PAN to use as a positive control along with WT. The percent input of PAN RNA from ORF59 HA-IPs, along with isotype control IgG-IPs and cellular GAPDH RNA control from ORF59 HA-IPs, was calculated using the Ct values from three biological replicates (Fig. 2B). Percent input was calculated for each sample based on a starting input fraction of 10%. WT and the ORF59 deletion mutant ORF59 Δ101–150, which showed an interaction with PAN by RT-PCR, had a similar percent input around 1%. However, the RT-qPCR for ORF59 Δ51–100, Δ251–300, and Δ351–396 indicated an almost complete loss of binding to PAN RNA, consistent with the results observed in the RT-PCR gel. These data suggest that ORF59-PAN RNA interaction is dependent on the 51–100, 251–300, and 351–396 amino acid regions of ORF59.

### PAN RNA forms aggregates when unable to interact with ORF59 during lytic replication

**Fig 3:**
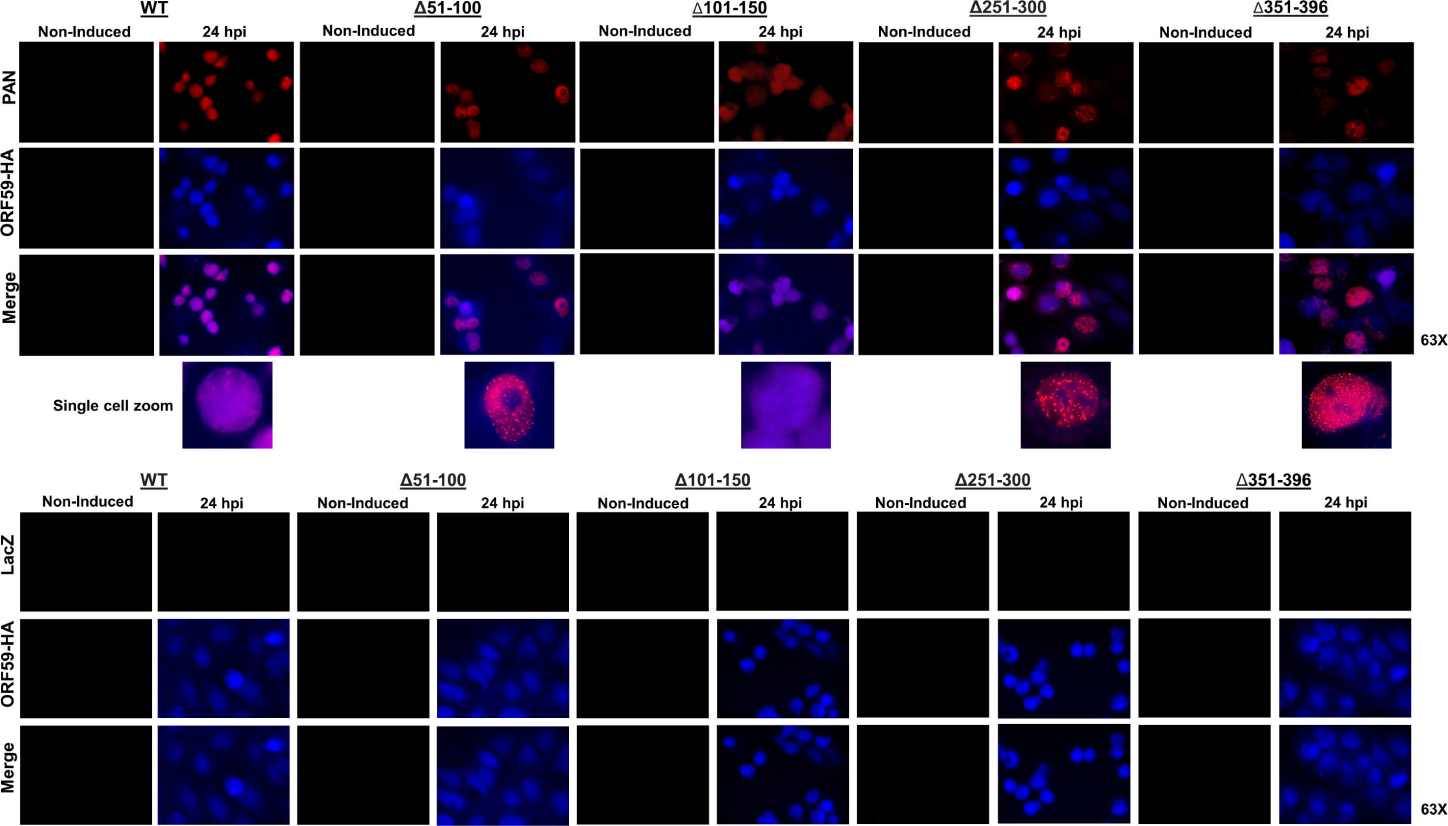
Localization of PAN RNA and ORF59 in iSLK ORF59HA-WT cells or ORF59HA deletion mutants. FISH was performed to determine the localization of ORF59 and PAN RNA in WT and ORF59 Δ51–100, Δ 251–300, and Δ 351–396 iSLK cell lines. 59HA-WT and deletion mutant cell lines were either non-induced or induced to undergo lytic reactivation with Dox, TPA, and NaB 24 hours before performing FISH and Immunofluorescence assay (IFA). For the FISH assay, biotinylated DNA probes complementary to PAN RNA or LacZ were hybridized, followed by streptavidin 594 to visualize PAN RNA (red). IFA was performed using an anti-HA antibody and secondary anti-rabbit 405 (blue) to detect ORF59HA. Coverslips were mounted with Antifade Gold and visualized using a fluorescent microscope (Zeiss). The ORF59 Δ101–150 iSLK cell line was used as a control for a deletion mutant of ORF59 which still retained the ability to interact with PAN RNA. The upper panels are PAN RNA FISH, and immediately below are zoomed images of the aggregate formations. The lower panel contains the control LacZ FISH.

Both ORF59 and PAN RNA localize to the nucleus during KSHV lytic replication. Our previous study showed no defect in ORF59 localization in any of the deletion mutants, including the mutant lacking the putative C-terminus NLS, suggesting other mechanisms or additional NLS used to localize the nucleus (19). We wanted to determine if PAN RNA exhibited any aberrant localization patterns in the ORF59 mutants unable to associate with PAN RNA (ORF59 Δ51–100, Δ251–300, and Δ351–396). In addition to the deletion mutants that lost interaction with PAN RNA, we chose one deletion mutant (ORF59 Δ101–150) that maintained its ability to interact with PAN RNA, which served as a positive control along with WT. Fluorescent *in situ* hybridization (FISH) was performed to determine the localization of PAN RNA along with immunofluorescence staining for ORF59 at 24 hours post reactivation with Dox, TPA, and NaB. Non-induced latent 59HA-WT containing the indicated BACmids that did not receive treatment with Dox, TPA, and NaB were used as controls. Biotinylated DNA probes complementary to PAN RNA (or LacZ as a control) were hybridized to the RNA followed by incubation with streptavidin 594 (Red) as previously described (41). To detect ORF59, samples were incubated with an anti-HA antibody followed by a secondary antibody with Alexa Fluor 405 (Blue). The 59HA-WT and ORF59 deletion mutants are derived from BAC16 which constitutively expresses green fluorescent protein (GFP). Consequently, the blue channel was used to detect ORF59, and a nuclear stain like DAPI could not be utilized, and therefore, the fluorescent images depicted in Fig. 3 illustrate the relative localization and patterns of PAN RNA (red) alongside ORF59 (blue) and not necessarily nuclear localization. In the cell lines which contain a deletion of ORF59 unable to interact with PAN RNA (ORF59 Δ51–100, Δ251–300, and Δ351–396 cell lines), we noted PAN RNA formed more aggregates compared to 59HA-WT or the ORF59 deletion (ORF59 Δ101–150) which still retained the ability to interact with PAN. Below the merged images is a panel containing zoomed images showing a closer look at the PAN aggregates in the ORF59 Δ51–100, Δ251–300, and Δ351–396 cell lines compared to controls. The disruption in the localization pattern of PAN RNA in the ORF59 Δ51–100, Δ251–300, and Δ351–396 suggests that those regions of ORF59 play a role in preventing PAN RNA from forming large aggregates during lytic replication.

### The 51-100 aa region of ORF59 is required for the interaction with ORF57

**Fig 4:**
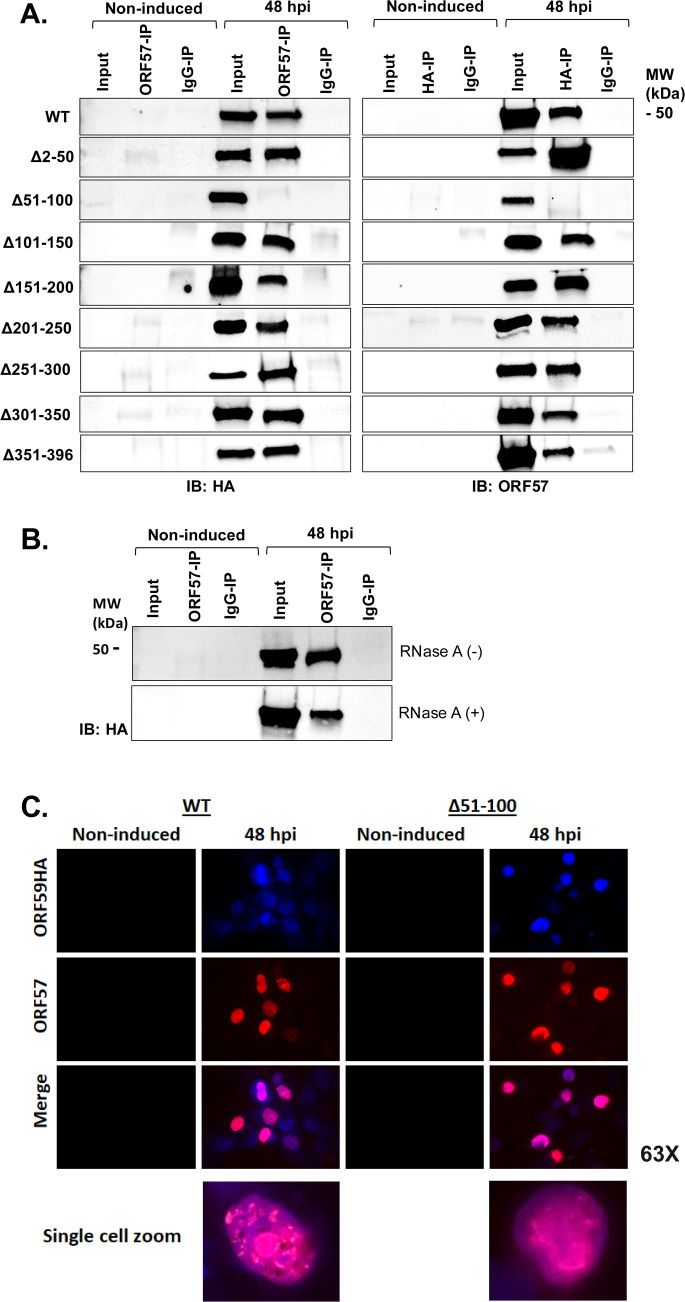
The 51–100 amino acids of ORF59 are required for interaction with the ORF57 protein. (**A**) Reciprocal co-IPs were performed from iSLK cell lysate from 59HA-WT or ORF59 deletion mutants induced with Dox, TPA, and NaB. Samples were harvested at 48 hpi, and IPs were performed with an anti-ORF57 antibody, anti-HA antibody (for ORF59), or IgG control antibody. Protein from input and IP samples were resolved by SDS-PAGE and western blots probed with either anti-HA or anti-ORF57 antibodies as indicated. Control samples are non-induced 59HA-WT cells, that were harvested along with the reactivated samples. (**B**) ORF57-IP from iSLK 59HA-WT lysate harvested 48 hpi. The lysate was divided, and half was treated with RNAse-A (indicated by a “+”) prior to the immunoprecipitation. Input and IP samples were resolved by SDS-PAGE, and western blots were probed with anti-HA antibody to visualize ORF59HA. Non-induced cells and IgG-IP were used as controls. (**C**) At 48 hpi, IFA was performed on 59HA-WT and ORF59 Δ51–100 iSLK cell lines to detect ORF59-HA using anti-HA antibody and anti-rabbit Alexa Fluor 405 (blue) secondary antibody, and ORF57 using anti-ORF57 antibody and anti-mouse Alexa Fluor 594 (red) secondary antibody. Coverslips were mounted and visualized by fluorescent microscopy (Zeiss). The GFP channel was not included as all cells containing KSHV BAC16 derivatives (including 59HA-WT and ORF59 Δ51–100) constitutively express GFP.

Next, we used the ORF59 deletion mutants to investigate the region of ORF59 which interacts with ORF57. Co-immunoprecipitations (co-IPs) were performed with 59HA-WT or ORF59 deletion iSLK cell lines reactivated with Dox, TPA, and NaB. Cells were lysed 48 hours post-induction (hpi). Reciprocal co-IPs were performed to target either ORF59 using an anti-HA antibody or ORF57 with an anti-ORF57 antibody. Due to the similar molecular weight between ORF59 protein and ORF57 protein (~50 kDa) and difficulty in interpreting re-probed blots, samples were split, and half of the lysate was used for co-IPs using anti-HA antibody and half using anti-ORF57 antibody. Antibody protein complexes were isolated with protein G magnetic beads, followed by extensive washing to remove non-specific complexes, and the eluates were assessed by SDS-PAGE western blots using antibodies to detect ORF59HA and ORF57 (Fig. 4A). The ORF59 Δ51–100 was unable to interact with ORF57, while all other deletion mutants, along with the 59HA-WT, showed an interaction between ORF59 and ORF57 (Fig. 4A).

Because ORF59 and ORF57 both interact with PAN RNA, we wanted to rule out the possibility that the interaction was mediated by the presence of PAN RNA. To accomplish this, we performed ORF57 co-IP experiments with the addition of RNase A to degrade all cellular and viral RNA within the lysate. The input lysate along with the ORF57-IP samples was resolved by SDS-PAGE, and the western blots were probed with anti-HA antibody. The ORF57 and ORF59 proteins retained the ability to interact in the samples treated with RNase A, revealing that no RNA is necessary for the interaction (Fig. 4B). However, this does not rule out that these three viral factors may act as a complex to perform certain functions during replication.

Lastly, we performed IFA at 48 hpi to assess the localization differences of ORF57 in 59HA-WT and ORF59 Δ51–100 cell lines (Fig. 4C). Unlike the aberrant PAN RNA localization (Fig. 3), we did not observe any substantial differences in the localization pattern of ORF57 when it was unable to interact with ORF59 in the ORF59 Δ51–100 mutant compared to 59HA-WT. These results suggest that ORF59-ORF57 interaction does not influence the localization of ORF57.

### Expression of a dominant negative polypeptide amino acids 30–100 of ORF59 disrupts the interaction between ORF59 and PAN RNA

**Fig 5:**
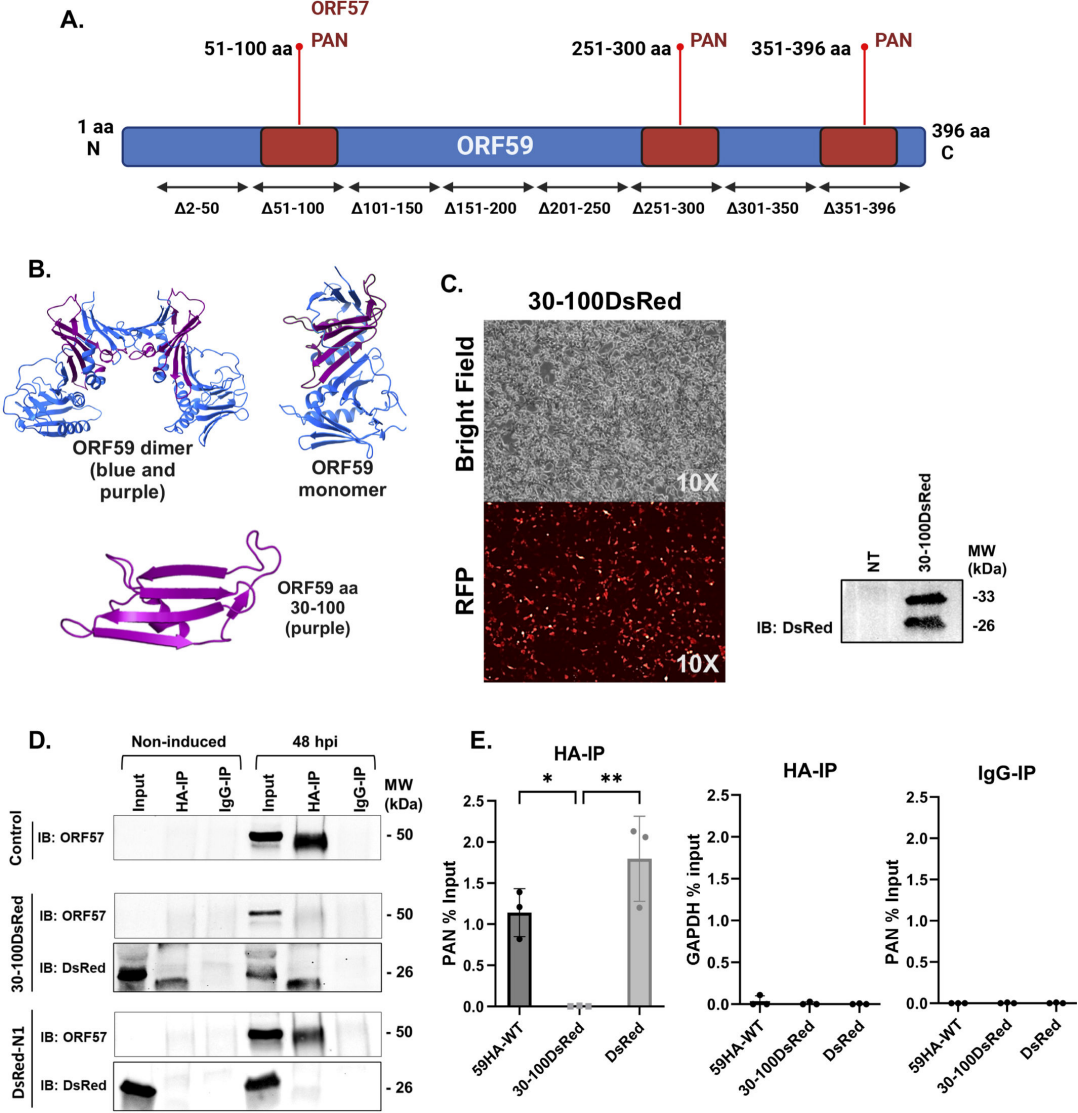
Expression of ORF59 aa 30–100 polypeptide during reactivation abrogates the interaction between full-length ORF59 and ORF57 and reduces the interaction between full-length ORF59 and PAN RNA. (**A**) The identified domains of ORF59 that interact with ORF57 and PAN RNA. (**B**) ORF59 protein structure (PDB ID: 3HSL) highlighting the aa 30–100 domain (magenta). The ribbon structures for ORF59 dimer and monomer are shown, along with the 30–100 polypeptide. (**C**) Mammalian expression vector was constructed to express ORF59 amino acids 30–100 with an in-frame DsRed tag (30–100DsRed). Cells were transfected with 30–100DsRed expression, and live cell images were captured 48 hours post-transfection. Bright Field and red fluorescence images were captured using Olympus CKX53 with pE-300lite at 10× magnification. Western blots were performed using lysate from NT control and cells transfected with the 30–100DsRed expression plasmid. Western blots were probed with anti-DsRed antibody. (**D**) The 59HA-WT and Δ51–100 iSLK cell lines were transfected with either pDsRed-NI vector (control) or 30–100DsRed expression plasmid. Co-IPs were performed following a 48-hour treatment with NaB, TPA, and Dox. ORF59HA and associated proteins were immunoprecipitated with an anti-HA antibody. Input and IP samples were resolved by SDS-PAGE and analyzed by western blots probed with either anti-HA (ORF59), anti-ORF57, or anti-DsRed antibodies overnight. (**E**) ORF59 RIPs were performed in 59HA-WT and 59HA-WT transfected with either pDsRed-N1 or 30–100DsRed expression plasmid. Protein-RNA complexes were isolated with anti-HA antibody and protein G beads. RT-qPCR was performed with primers/probes specific for PAN RNA. Percent input was calculated, and error bars are the SD from three biological replicates. An unpaired *t*-test was performed, *P = 0.05 and **P = 0.0025.

Given that the 51–100 aa region of ORF59 is necessary for the interaction with both PAN RNA and ORF57, we wanted to determine the effects of introducing a small polypeptide containing that domain into 59HA-WT iSLK cells prior to lytic reactivation. A mammalian expression vector was constructed to express ORF59 amino acids 30–100 along with a C-terminal DsRed tag (named 30–100DsRed). We extended the designed region to include amino acids 30–100 rather than solely 51–100, considering the planar surface formed by the inclusion of the 20 upstream amino acids, comprising four complete beta-sheets (Fig. 5A). We hypothesized that by including these additional beta sheets, the small polypeptide would exhibit greater stability and correct folding. Specifically, while 51–100 alone generates the two outer beta sheets, the 30–50 amino acids contribute to the formation of the inner beta sheets. After generating the plasmid, we transfected the 30–100DsRed expression plasmid into 59HA-WT iSLK cells. We observed DsRed expression in the live cells (Fig. 5B). To further confirm the expression of the 30–100DsRed plasmid, western blot analysis of the protein lysate was performed 48 hours post-transfection. A non-transfected (NT) set was used as a negative control. We noted a doublet band in the 30–100DsRed lane on the western blot, the predicted size of the polypeptide is 33 kDa, and the smaller band may represent a product from a cryptic start site or post-translational cleavage. After confirming the expression of DsRed tagged 30–100 polypeptide, co-IPs were performed in 59HA-WT iSLK cells transfected with either the pDsRed-N1 vector alone (control) or the 30–100DsRed expression plasmid followed by a 48-hour treatment with Dox, TPA, and NaB. ORF59HA protein complexes were isolated with anti-HA antibody and protein G beads followed by extensive washing as previously described. The proteins from input and IP samples were analyzed by western blots incubated with either anti-ORF57 or anti-DsRed antibodies (Fig. 5C). In cells that were transfected with the 30–100DsRed polypeptide expression plasmid, the IP of ORF59 did not interact with ORF57, while in the control cells, DsRed-N1 ORF57 still interacted with ORF59. This suggests that the 30–100DsRed was able to block the interaction between Full-length ORF59 and ORF57 during lytic replication.

To test the dominant negative effect of the ORF59 30–100 polypeptide on the association between PAN RNA and ORF59, we performed RIP assays from 59HA-WT, 59HA-WT transfected with 30–100DsRed, or transfected with DsRed-N1. Percent input was calculated and graphed with SD error bars from three biological replications (Fig. 5D). We measured a significant decrease in the amount of PAN RNA associated with ORF59 in the presence of 30–100DsRed compared to 59HA-WT and the DsRed-N1 vector control. GAPDH percent of input and PAN RNA percent of input in the IgG-RIP were performed as a cellular and isotype negative control, respectively.

### ORF59 aa 30–100 acts as a dominant negative inhibitor to disrupt viral DNA accumulation and infectious virus production

**Fig 6:**
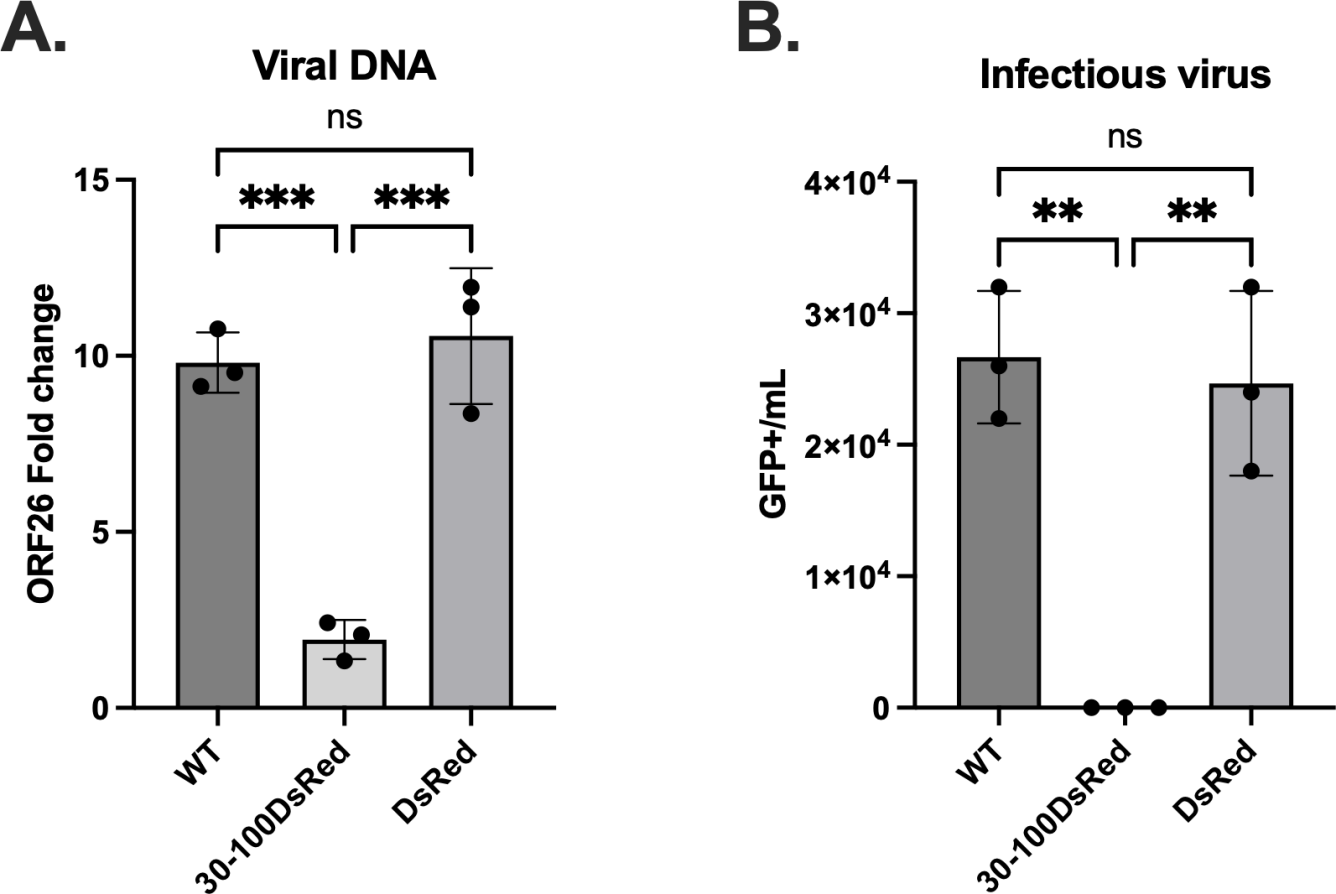
The ORF59 aa 30–100 acts as a dominant negative polypeptide to disrupt viral DNA accumulation and infectious virus production. iSLK cell lines containing 59HA-WT were transfected with either pDsRed-N1 vector or 30–100DsRed expression plasmid 24 hours before reactivation with Dox, TPA, and NaB. (**A**) DNA accumulation of 59HA-WT, 59HA-WT transfected with pDsRed-N1 vector, or 30–100DsRed was measured at 72 hpi by qPCR with KSHV-specific primers/probes (ORF26). Relative viral DNA was normalized with cellular DNA, and fold change was calculated using non-induced controls. Statistical analysis was performed using a one-way ANOVA with Tukey’s multiple comparisons, and results represent three biological replicates ***P = 0.0003. (**B**) Quantification of infectious viruses from iSLK cell lines containing 59HA-WT and transfected with either pDsRed-N1 vector or 30–100DsRed expression vector. The virus was harvested from cells and supernatant at 5 dpi. A 10-fold serial dilution of the inoculum was used to infect 293L cells, and GFP+ foci were counted at 3 dpi. Statistical analysis was performed using a one-way ANOVA with Tukey’s multiple comparisons, and results represent data from three independent experiments **P = 0.001.

Finally, we assessed the 30–100DsRed ORF59 polypeptide’s ability to inhibit viral replication by measuring viral DNA accumulation and infectious virus production. To test the effect of the 30–100DsRed peptide on WT replication, the 59HA-WT or cells transfected with 30–100DsRed or DsRed-N1 vector alone were induced with Dox, TPA, and NaB for 72 hours along with uninduced controls. Total DNA was harvested with DNA lysis buffer followed by phenol-chloroform extraction and measured by qPCR. KSHV-specific primers/probes (ORF26) were used to quantify viral DNA and were normalized to the cellular DNA control (7SK). By 72 hours post-induction, there was a significant decrease, approximately fivefold reduction, in the amount of viral DNA accumulation in the cells that were transfected with the 30–100DsRed expression plasmid compared to the control cells (Fig. 6A). There was no substantial difference in viral DNA replication for the cells transfected with DsRed control compared to the non-transfected WT cells. These results suggest that the 30–100DsRed can act to block early replication events.

After measuring a significant reduction in DNA accumulation with the addition of the 30–100DsRed expression plasmid, we examined the effects on infectious virus production. Virus inoculum was obtained from 59HA-WT cells plated at 5 × 10^6^ seed density and transfected with either 30–100DsRed or DsRed-N1 vector and induced with Dox, TPA, and NaB. At 5 days post-induction, virus was harvested from cells and media supernatant. The amount of infectious virus was determined by serial dilution of the inoculum onto 293L cells plated at 2.5 × 10^5^. After 72 hours, GFP foci were counted to calculate the amount of infectious virus. Plots generated are GFP+ cells/mL (Fig. 6B). The average amount of GFP+ cells/mL measured in the WT alone was 2.67 × 10^4^, with DsRed control was 2.47 × 10^4^, and in the presence of the 30–100 DsRed, we measured no GFP foci. In the cells transfected with 30–100DsRed, virus production was significantly reduced compared to the 59HA-WT and DsRed control.

## DISCUSSION

DNA viruses, such as KSHV, replicate within the nuclei in specialized areas called viral replication compartments (42, 43). In these compartments, cellular and viral proteins and RNAs are recruited to form complexes for efficient replication (42, 43). This investigation focused on protein-protein (ORF59-ORF57) and protein-RNA (ORF59-PAN RNA) interactions during lytic reactivation to determine if these interactions are required for successful replication. The individual functions of ORF59, ORF57, and PAN RNA have previously been explored, but the functions of these protein-protein and protein-RNA complexes have not been fully explored. We hypothesized that the interactions of ORF59 protein with ORF57 protein and PAN RNA are necessary for virus replication and together promote the coupling of transcription and RNA stability with nascent DNA synthesis.

The first objective of this study was to determine the amino acid domains of ORF59 that interact with ORF57 and PAN RNA. To investigate this, we used a series of ORF59 deletion mutants in iSLK BAC16 cells and performed co-IPs and RIP assays during KSHV reactivation. We identified amino acids 51–100, 251–300, and 351–396 of ORF59 associated with PAN RNA, and amino acids 51–100 interact with ORF57. We also use FISH and IFA to assess a localization difference in ORF57 and PAN RNA in the ORF59 mutants that no longer interacted with these factors. Interestingly, we did not observe any defect in the ORF57 localization. However, in the three ORF59 deletion mutants that were no longer associated with PAN RNA, we found concentrated aggregates of PAN RNA compared to a homogeneous diffuse pattern in WT and ORF59 mutants that still retained the ability to interact with PAN. This observation suggests that ORF59 may play a role in keeping PAN RNA diffuse within the nucleus. We acknowledge that there are limitations to using internal deletion mutants of ORF59 to identify interaction domains, and the deletions may change the conformation of the surrounding regions, which could indirectly influence the interactions.

Once we identified the 51–100 region of ORF59 as being required for the interaction for both ORF57 and PAN RNA, we used that information to create a small polypeptide containing amino acids 30–100 of ORF59 to act as a dominant negative and disrupt the interactions with full-length ORF59. When the 30–100 aa was introduced into 59HA-WT iSLK cells before lytic reactivation, the result was a decrease in infectious virus production. We hypothesize that the 30–100 aa region of ORF59 acts as a dominant negative inhibitor of virus replication. By definition, a dominant negative inhibitor is a polypeptide, often a mutant or truncated form of the wild-type protein, that, when overexpressed, disrupts the activity of the wild-type factor (44). The 30–100 polypeptide impairs the function of the full-length protein, in this case ORF59, by blocking its binding to ORF57 and PAN RNA. This demonstrates the functionality of the 51–100 amino acid region of ORF59 specifically in its capacity for protein-protein and protein-RNA interactions.

In addition to previously identified regions of ORF59 required for histone binding, DNA polymerase interaction, nuclear localization, and phosphorylation, in this study, we have identified that the 51–100, 251–300, and 351–396 aa domains of ORF59 support additional interactions. Determining the exact functional role of the domains of ORF59 is complicated by the overlapping interactions that often occur. Our strategy was to identify the domains of ORF59 that mediate the interaction with ORF57 and PAN RNA and to use that information to create a dominant negative peptide that would specifically target those interactions. We decided to go forward with a peptide that encompasses the 51–100 amino acid area since it was the binding domain for both ORF57 and PAN RNA. Co-IPs for ORF59-ORF57 and RIPs for ORF59-PAN RNA demonstrated that both interactions were disrupted in the presence of the 30–100 polypeptide. We acknowledge the possibility that the lack of PAN RNA binding could be indirect through its association with ORF57. Surprisingly, in the presence of the 30–100 polypeptide, we measured an almost complete lack of association with PAN RNA and ORF59 by RIP; however, the other identified domains (aa 251–300 and 351–396) associating with PAN RNA are still present. We hypothesize that the 30–100 polypeptide has a higher affinity for PAN RNA than aa 251–300 or 351–396, so there may be relatively little PAN available to interact with the other domains. Additionally, we did not test if the 30–100 peptide disrupts the ability of ORF59 to homodimerize which may also be required for the interaction with ORF57 and PAN RNA. The known dimerization domains are within 1–21 aa and 277–307 aa, so although the 30–100 peptide is outside of these regions, and unlikely to disrupt the dimerization, it is still a possibility.

There are some limitations to our study. First, although the 30–100 aa polypeptide was created to specifically target the interaction between full-length ORF59 and ORF57 or PAN RNA, we cannot rule out the possibility that this domain interferes with the classical function of ORF59 as a processivity factor during DNA replication. In addition, the 30–100 aa may have off-target effects which could lead to an indirect reduction in virus replication. Because we note a significant reduction in DNA accumulation, it is difficult to determine if the mechanism of action for the 30–100 polypeptide is directly acting on DNA synthesis itself or secondary to other defects such as viral transcription or RNA stability. Further study will need to be done to determine the exact mechanism and functions of the interactions between ORF59, ORF57, and PAN RNA. Since we observed a significant decrease in viral DNA synthesis, we hypothesize that interaction between ORF59, ORF57, and PAN RNA begins during early times, but further assessment of IE, E, and L transcripts will need to be performed to properly evaluate the time course of events related to the interactions. Additionally, we only tested the dominant negative peptide in KSHV-infected iSLK cells, and further studies will need to be performed in other models of KSHV infection, such as *de novo* infection in endothelial cells or reactivation in BCBL-1, to determine if the dominant negative can hinder replication in those models of KSHV infection similar to the iSLK model. The reduction in virus production with the 30–100 aa polypeptide is an exciting possibility and demonstrates that targeting viral processivity factor protein-protein and protein-RNA interactions may be useful for antiviral therapeutics.

## MATERIALS AND METHODS

### Cell lines

Human HEK293L (RRID: CVCL_M775) and HEK293FT (RRID: CVCL_6911) were cultured and maintained in antibiotic-free Dulbecco’s modified Eagle medium (DMEM; Corning, cat. # 45000–304) supplemented with 10% fetal bovine serum (Corning, Ref. # 35–010-CV). 293L cells were obtained from the National Institutes of Health (NIH) AIDS reagent program. iSLK cells were maintained in DMEM with 10% fetal bovine serum 1 µg/mL of puromycin and 250 µg/mL of G418 (InvivoGen). iSLK cells containing the KSHV bacterial artificial chromosome 16 (BAC16) were maintained in identical conditions with the addition of 1.2 µg/mL hygromycin B (U.S. Biologicals). To induce lytic reactivation in the described cell lines, 0.25 mM NaB (Sigma-Aldrich), 1 µg/mL Dox (U.S. Biologicals), and 10 ng/mL of TPA (Sigma-Aldrich) were administered at 24 hours post-plating. Cell lines were grown and maintained at 37°C in a humidified incubator supplemented with 5% CO_2_.

### Plasmids

The EcoRI restriction enzyme (New England Biolabs #R0101M) was used to linearize the phCMV-XI (pXI) vector for ORF59_30–100DsRed plasmid generation. The ORF59_30–100 sequence was synthesized as gBlocks (IDT) with the final construct containing an in-frame DsRed tag. The homologous regions used to target the pXI vector during cloning are underlined, and the DsRed tag is indicated by bold font: gcttcgaattccaccATGAAAACCGGAGTAGTGCAAGTGCACGGATCGGCTTGCACGCCAACCCTCAGTGTGCTGTCCAGCGTGGGGACAGCTGGCGTTCTGGGGTTAAGAATAAAGA**ATGCCCTTACGCCCCTGGTGGGACACACGGAAGGCAGTGGAGACGTTAGCTTCAGCTTCAGGAATACGTCCGTCGGTAGCGGCTTCACGCACACGCGTGAGCTATTCGGTATGGTGCGCTCCTCCAAGAACGTCATCAAGGAGTTCATGCGCTTCAAGGTGCGCATGGAGGGCACCGTGAACGGCCACGAGTTCGAGATCGAGGGCGAGGGCGAGGGCCGCCCCTACGAGGGCCACAACACCGTGAAGCTGAAGGTGACCAAGGGCGGCCCCCTGCCCTTCGCCTGGGACATCCTGTCCCCCCAGTTCCAGTACGGCTCCAAGGTGTACGTGAAGCACCCCGCCGACATCCCCGACTACAAGAAGCTGTCCTTCCCCGAGGGCTTCAAGTGGGAGCGCGTGATGAACTTCGAGGACGGCGGCGTGGTGACCGTGACCCAAGACTCCTCCCTGCAGGACGGCTGCTTCATCTACAAGGTGAAGTTCATCGGCGTGAACTTCCCCTCCGACGGCCCCGTAATGCAGAAGAAGACCATGGGCTGGGAGGCCTCCACCGAGCGCCTGTACCCCCGCGACGGCGTGCTGAAGGGCGAGATCCACAAGGCCCTGAAGCTGAAGGACGGCGGCCACTACCTGGTGGAGTTCAAGTCCATCTACATGGCCAAGAAGCCCGTGCAGCTGCCCGGCTACTACTACGTGGACTCCAAGCTGGACATCACCTCCCACAACGAGGACTACACCATCGTGGAGCAGTACGAGCGCACCGAGGGCCGCCACCACCTGTTCCTG**tagggctaagaattctgcagtcgacggta. The ORF59_30–100DsRed insert was cloned into pXI vector and transformed using GeneArt Seamless Cloning and Assembly (Thermo Fisher Scientific) followed by transformation in One Shot TOP10 chemically competent *Escherichia coli* (cat. C404010) according to the manufacturer’s protocol. The pDsRed-N1 (Clontech) was used as a control plasmid for DsRed expression alone. Plasmid constructs were sequenced by Sanger sequencing conducted at Azenta/GENEWIZ (Azenta US, Inc., South Plainfield, NJ, USA).

### Plasmid transfection

Transfections were completed using TransIT-LT1 Transfection Reagent (MIR 2300 Mirusbio) and Opti-MEM Reduced Serum Medium (31985062 Gibco) following the manufacturer’s instructions. Briefly, 18 hours post-plating, cells were transfected with either 30–100DsRed or DsRed-N1 following the guidelines in the TransIT-LT1 protocol. Cells were then incubated for 48 hours, or 24 hours, and induced following the above-listed conditions for the specified times (0.25 mM NaB, 1 µg/mL Dox, and 10 ng/mL TPA).

### RNA immunoprecipitation

For RIP, iSLK BAC16-59HA WT (59HA-WT) cells were plated in 10 cm dishes at 6 × 10^5^ seed density. The same was true for the 51–100 deletion and the WT transfected with either 30–100DsRed or DsRed-N1 alone. For transfected cells, induction was done 24 hours post-transfection. All cells were induced for 24 hours (0.25 mM NaB, 1 µg/mL Dox, and 10 ng/mL TPA). At the end of the induction period, the media was removed, and cells were washed with ice-cold phosphate-buffered saline (PBS). Crosslinking was performed with a 1% solution of formaldehyde in PBS and incubated for 10 minutes at room temperature. A total of two PBS washes were done before adding a solution of 250 mM glycine in PBS for 5 minutes at room temperature. The samples were washed an additional two times with ice-cold PBS. To lyse cells, 1 mL of IP lysis [Pierce, Thermo Fisher IP lysis buffer, 1:100 proteinase inhibitor (Sigma-Aldrich), 1:300 RNase Out (Invitrogen), and 1 mM phenylmethylsulphonyl fluoride (Sigman-Aldrich)] was incubated on ice for 5 minutes. Samples were sonicated for 10 pulses of 20 seconds with a probe disruptor (Misonix 200; Thermo Fisher). After sonication, samples were cleared by centrifugation at 800 × *g* for 5 minutes at 4°C. A solution containing 1% bovine serum albumin (BSA), 10 µg/mL of yeast RNA, and 1 mL IP Lysis Buffer was used to block Dynabeads Protein G magnetic beads (Thermo Fisher, Invitrogen, Catalog number: 10003D) for 30 minutes at room temperature. Beads were subsequently washed twice with IP Lysis Buffer and resuspended. A total of 100 µL input was removed and kept at −80°C, and the remaining 900 µL was used for the IP reaction. Protein G beads were incubated with anti-ORF57-Mouse (Santa Cruz) or anti-HA-Rabbit (Sigma Aldrich) antibodies at 1:200 concentration for 1 hour before lysate was added. Samples were then incubated overnight rotating at 4°C.

Following incubation, beads were washed a total of five times. A low-salt wash [0.1% SDS, 1% Triton X-100, 2  mM EDTA, 20  mM Tris-HCl (pH 7), and 150  mM NaCl], high-salt wash [0.1% SDS, 1% Triton X-100, 2  mM EDTA, 20  mM Tris-HCl (pH 7), and 500  mM NaCl], LiCl wash [0.25 M LiCl, 1% NP-40, 1% Na-deoxycholate, 1  mM EDTA, and 10  mM Tris-HCl (pH 7)], and two 1 × Tris EDTA (TE) washes were done in sequential order. The final TE wash was removed from the beads, and 200 µL reverse buffer [10 mM Tris-HCl (pH 7), 5  mM EDTA, 10  mM dithiothreitol, and 1% SDS) and 100 µL proteinase K solution [10 mM Tris-HCl (pH 7), 1 mM EDTA, 0.5% SDS, 100 mM NaCl, and 5% proteinase K] was added to the beads. In contrast, 100 µL of each was added to the input. Samples were then incubated for 1 hour at 42°C followed by 1 hour at 65°C.

RNA was extracted using Trizol LS (Thermo Fisher, Life Technologies). Briefly, 900 µL of Trizol LS was added to each sample and incubated for 5 minutes at room temperature. Chloroform isoamyl was added (20%, 240 µL), mixed by vortex for 15 seconds, and incubated for 15 min at room temperature. Samples were centrifuged at 4°C at 13,000 × *g* for 10 minutes. To new tubes, 650 µL of supernatant was transferred and RNA precipitated with isopropanol and Glycoblue. After a 15-minute incubation, samples were centrifuged at 13,000 × *g* at 4°C for 10 minutes. The supernatant was discarded, and the pellet was washed with 1 mL 75% ethanol and centrifuged for 5 minutes at 7,500 × *g*. The pellet was then air dried and resuspended in 36 µL H_2_O and RNase Out solution.

DNA was removed from extracted RNA using the Turbo DNA-free kit (Thermo Fisher). Lastly, cDNA was generated using iScript reverse transcription supermix for RT-qPCR (Bio-Rad). PCR was performed using Prime Star Max (TaKaRa) with primers specific for PAN and U1 with the following primers: PAN forward: 5′-TTGGCCTGAGAGCTGTAGTA-3′, PAN reverse: 5′-CACAGAACCGAAACAACGAATG-3′, U1 Forward: 5′-ATACTTACCTGGCAGGGGAG-3′, and U1 Reverse: 5′-CAGGGGAAAGCGCGAACGCA-3′. Samples were run on Tris, acetate, and EDTA 1% agarose gel with 10 µL of each PCR product. For the RT-qPCR assay, the following TaqMan primers and probes (IDT) were used.

**Table:** 

Primers used for qPCR	Primer 1	Primer 2	Probe
GAPDH	5′-ACA TCG CTC AGA CAC CAT G-3′	5′-TGT AGT TGA GGT CAA TGA AGG G-3′	5′−56-FAM/AA GGT CGG A-Zen-G TCA ACG GAT TTG GTC /3IABkFQ-3′
7SK	5′-TGA CTA CCC TAC GTT CTC CTA C-3′	5′-GTC AAG GGT ATA CGA GTA GCT G-3′	5′−56-FAM/CC CTG CTA G-Zen-A ACC TCC AAA CAA GCT /3IABkFQ/
ORF26	5′-ATG GCA CTC GAC AAG AGT ATA G-3′	5′-GGC AGT ACG CTC CCT ATT T-3′	5′–56-FAM–AGA CTC TTC–ZEN–GCT GAT
PAN	5′-CGATTTACACTCAATCCGCTTTC-3′	5′-GTGCACTACCTATCTGCTCATT-3′	5′–56-FAM–CGTTGTTTC–ZEN–GGTTCTGTGTTTGTCTGA–3IABkFQ–3′

### Western blotting and co-IP

Cells were plated in 10 cm dishes at 6 × 10^5^ seed density. For experimental sets, cells were induced with 0.25 mM NaB, 0.5 µg/mL Dox, and 10 ng/mL TPA for 48 hours. Total protein lysate was prepared following three washes with ice-cold PBS. A total of 1 mL IP lysis buffer (Pierce, Thermo Fisher) and protease inhibitors (1:100; catalog number P8340; Sigma-Aldrich) were added to each dish and lysed for 10 minutes on ice, rocking. Lysates were then sonicated using four 10-second pulses with a probe disruptor (Misonix 200; Thermo Fisher) and centrifuged (13,000 × *g* for 10 minutes at 4◦C). For input controls, 100 µL was combined with 100 µL of 2× Laemmli buffer (Bio-Rad cat # 161–0737) with β-mercaptoethanol and boiled for 5 minutes at 95°C. Inputs were stored at −20°C until it was run on an SDS-PAGE gel with the immunoprecipitation samples.

Samples were rotated with anti-ORF57 (Santa Cruz) or anti-HA (Sigma Aldrich) antibodies overnight at 4°C. Additionally, samples containing either mouse or rabbit anti-IgG were included as isotype control. Following incubation, magnetic protein G beads were added (1:10) and rotated at room temperature for 2 hours. Immunoprecipitants were collected by centrifuging at 6,000 × *g* for 1 minute at 4°C. The supernatant was aspirated and discarded without disturbing the beads. The beads were washed three times for 5 minutes with IP lysis buffer. After the final wash, the supernatant was aspirated, discarded, and beads resuspended with 100 µL of 2× Laemmli buffer with β-mercaptoethanolnd boiled for 5 minutes at 95°C.

Western blotting was performed as previously described (41). Briefly, 30 µL of protein was resolved by SDS-PAGE gel and subsequently transferred to a polyvinylidene difluoride membrane (Bio-Rad). Blots were blocked for 15 min with 5% skim milk in Tris-buffered saline with 0.1% Tween 20 [TBST; 10 mM Tris-HCl (pH 7.5), 150  mM NaCl, and 0.05% Tween 20] and then incubated with the following specific antibodies: rabbit anti-HA at 1:2,000 (catalog number H6908; Sigma-Aldrich), mouse anti-ORF57 at 1:400 (catalog number SC-135746; Santa Cruz Biotechnology), rabbit anti-DsRed at 1:1,000 (catalog number 632496, Living Colors, Takara), and mouse or rabbit IgG at 1:2,000. All primary antibodies were incubated at 4°C overnight, followed by washing with TBST and incubation with either IR-dye 680 and 800 anti-mouse or anti-rabbit (Li-Cor) secondary antibody for 30  minutes, and proteins were visualized using a ChemiDoc MP imaging system (Bio-Rad).

### Viral DNA quantification using qPCR

iSLK BAC16-59HA WT cells were plated at 2.5 × 10^5^ seed density. Samples containing WT induced, WT transfected with 30–100DsRed (5 µg), and WT transfected with pDsRed-N1 (5 µg) were induced for 72 hours. A non-induced set was used as a control. Cells were washed twice with ice-cold PBS and lysed with DNA extraction buffer containing 2% SDS, 10 mM Tris-HCl pH 7.4, and 10 mM EDTA. The cell lysate was spun briefly before adding 50 mg/mL of proteinase K. In a 65°C water bath, samples were incubated for 1–2 hours.

To extract DNA, 400 µL of phenol-chloroform was added to total cell lysate. Samples were thoroughly vortexed and spun at 13,000 × *g* for 2 minutes. The top layer was transferred to a new tube, and the process was repeated with chloroform-isoamyl. DNA was precipitated using 1/10th volume (40 µL-cold) of 3M sodium acetate and 2.5× (1.1 mL-cold) volume of 100% EtOH. DNA was pelleted by centrifugation at 13,000 × *g* for 15 minutes at 4°C. The supernatant was discarded, and DNA was washed with 500 µL of 70% EtOH. A second spin was performed at 13,000 × *g* for 10 minutes at 4°C. The supernatant was discarded, and the pellet was air-dried before being resuspended in 1 × TE (IDT, pH 7.5).

DNA was analyzed by qPCR as previously described (19, 41). Briefly, SsoAdvanced Universal Probes Supermix (Bio-Rad) and primer/probes made from IDT were used according to the manufacturer’s instructions. KSHV-specific primers (ORF26) were used to quantitate viral DNA synthesis and were normalized to cellular 7SK, the TaqMan primer/probe sequences used are indicated in the previous section. Each experiment was performed in triplicate, fold change was calculated from the non-induced samples, and the data shown are the average with SD (19, 41).

### Fluorescent *in situ* hybridization with immunofluorescence assay

In a 12-well dish with coverslips, iSLK BAC16-59HA WT, Δ51–100, Δ101–150, Δ251–300, and Δ351–396 were plated at 60,000 cells/well. Cells were either not induced (control) or induced (0.25 mM NaB, 1 µg/mL Dox, and 10 ng/mL TPA) for 24 hours. Samples were fixed with 4% paraformaldehyde in PBS for 30 minutes and washed three times before being permeabilized with 0.5% Triton for 10 minutes. For prehybridization, cells were washed with 2× Saline-Sodium Citrate (SSC) and treated with 1 mL of prehybridization buffer (50% formamide, 10% dextran sulfate, 2× SSC, 0.1% BSA, 500 µg/mL sheared salmon sperm DNA, 125 µg/mL yeast RNA, and 1 mM vanadyl ribonucleoside complexes) for 1 hour at 37°C.

PAN-Biotin oligo single-stranded DNA (ssDNA) probes were made into a cocktail containing 10 pmol of each of the 20 oligos as previously described. To each well, 1 mL of hybridization buffer with probes of PAN or LacZ (control) was added, and the plates were incubated overnight at 37°C in a humidity chamber. Following hybridization, cells were washed twice with 1× SSC for 10 minutes at 25°C and fixed with 4% paraformaldehyde before being permeabilized for 10 minutes with 0.5% Triton in PBS at 4°C. Samples were then prepared following the IFA protocol.

The IFA portion of the FISH/IFA was performed following permeabilization. Samples were first blocked with 3% goat serum for 30 minutes at room temperature, rocking. The primary antibody (2.5 µg/mL HA-Rabbit and 200 µL/well) was incubated for 2 hours, rocking. The primary antibody was removed, and samples were washed thrice with 1% BSA. The secondary antibody (1:500, anti-Rabbit 405, 200 µL/well) was incubated for 30 minutes at room temperature, rocking. The secondary antibody was removed, and samples were washed thrice with 1% BSA. Cells were then incubated with streptavidin-594 (25 µg/mL, 200 µL/well) in 0.1% BSA-PBS for 1 hour at room temperature, rocking. Wells were rinsed three times with PBS, and coverslips were mounted with prolonged antifade gold.

### Infectious virus assay

Virus inoculum was obtained from iSLK BAC16 ORF59-HA WT cells plated at 5 × 10^6^ seed density. The 59HA-WT and cells transfected with either pXI_ORF59_30–100DsRed or pDsRed-N1 alone were induced (0.25 mM NaB, 1 µg/mL Dox, and 10 ng/mL TPA) and incubated at 37°C with 5% CO_2_. At 5 days post-induction, cells and cell culture supernatant were harvested and frozen at −80°C followed by thawing at 37°C three times to lyse. Clarified media was obtained from three sequential spins of 10 minutes at 4,000 × *g*. Viruses were pelleted by centrifugation at 28,000 × *g* for 90 minutes in an SW28 rotor. The virus pellet was resuspended in 0.5  mL of antibiotic-free medium and serially diluted. 293L cells plated at 2.5 × 10^5^ were infected, and GFP foci were counted at 72 hpi. Plots generated are GFP (+) cells/mL.

### Statistical analysis

Unless indicated, statistical analyses were completed by one-way analysis of variance with multiple comparisons or unpaired student’s *t*-test. All statistical analyses were completed using GraphPad Prism software.
